# Association of American Medical Colleges

**Published:** 1878-07

**Authors:** 


### Association of American Medical Colleges.

### MORNING SESSION.

The Association of American Medical Colleges, organized at Chicago last year, met in the hall of the Buffalo Medical College, corner of Main and Virginia streets, at ten o'clock Monday morning, June 3d. The President, Prof. J. B. Biddle, of the Jefferson Medical College, presided, and the Sectetary, Dr. L. Connor, of the Detroit Medical College, performed the usual duties of that office.

Credentials were presented from the following named delegates from various medical colleges:

Rush Medical College—Prof Moses Gunn.

Jefferson Medical College—Profs. S. D. Gross and J. B. Biddle.

Medical Department University of Louisville—Prof. J. M. Bodine.

Detroit Medical College—Profs. L. Connor and E. W. Jenks.

Chicago Medical College—Prof. N. S. Davis.

Miami Medical College—Prof. W. H. Mussey.

Starling Medical College—Prof. H. G. Landis.

Medical Department of University of Nashville and Vanderbilt—Profs. T. Menees and W. T. Briggs.

Louisville Medical College—Prof. A. B. Cook.

Medical Department University of Iowa—Profs. E. F. Clapp, W. F. Peck, W. S. Robertson.

Kansas City College of Physicians and Surgeons—Prof. T. B. Lester.

Medical Department of Michigan University.—Prof. E. S. Dunster and George E. Frothingham.

Missouri Medical College—A. P. Lantford.

Bellevue Hospital Medical College—Prof. A. Flint, Jr.

Cleveland Medical College—Prof. X. C. Scott.

Objections to the admission of Howard University, D. C., to the Association, were taken up and discussed. The objections were sustained, and the University refused admission by a vote of 12 to 3.

The Secretary presented a report, from which it is learned that there are twenty-five regular members and one affiliated member. Applications for membership had been received from the Ohio Medical College March 26th, and the Alabama Medical College March 18th last. As soon as the report was issued last Fall a letter was sent to all regular Medical Colleges of the United States, asking if they conformed to the articles of confederation required of regular or affiliated members. Accompanying this letter was sent the pamphlet containing a history of the organization of the Association, its constitution, by-laws, articles of confederation, and list of members. Two colleges—the Medical Department of Harvard and the Medical Department of the University of Pennsylvania—replied that they regarded it unadvisable for them to join the Association. Seven hundred copies of the constitution, by-laws, etc., of the Association were printed and about half of them distributed to members, other colleges, *Medical Journals*, etc. The remainder are on hand at the disposal of the Association.

The following named Colleges have filed with the Secretary copies of the laws under which they were incorporated: College of Physicians and Surgeons, New York; Medical Department of University of Louisville, Louisville Medical College, Kentucky School of Medicine, Chicago Medical College, Bellevue Medical College, Detroit Medical College, Galveston Medical College and Hospital, Fort Wayne Medical College, and Starling Medical College.

An amendment to the constitution was offered by the Bellevue Hospital Medical College, regulating the number on which the fees shall be rebated, and another directing each College to make out a correct list of its matriculants. These were seconded by the College of Physicians and Surgeons of New York.

The annual reports from the following named Medical Colleges were received and read by the Secretary: Chicago Medical College, Medical Department University of Louisville, Detroit Medical College, Louisville Medical College, Medical Department University of Iowa, Missouri Medical College, Miami Medical College, Medical Department University of Wooster, Jefferson Medical College, College of Physicians and Surgeons of New York, Kansas City College of Physicians and Surgeons, Hospital College of Medicine, Louisville, Cleveland Medical College, Dartmouth Medical College, Galveston Medical College, Bellevue Hospital

Medical College, and the Michigan University. These reports noted the honorary degrees conferred by each college within the year, with the age and name of the recipient, and why the degree was given. They also gave the names of all persons who had been allowed remissions and reductions of established fees with the reasons in each case for the proceeding. The report of the Medical Department of the Wooster University noted the fact that the Honorary Degree had been conferred upon an individual twenty-eight years old. As the articles of confederation distinctly state that this degree shall not be conferred upon men under forty years of age, it was decided that the Secretary of the Association be directed to inform the College in question of its violation of the regulation noted, and ascertain its reasons therefor.

Prof. Flint offered a resolution, which, after considerable discussion between Profs. Peck, Davis, Dunster, Cook, Frothingham and others, was referred to a committee consisting of Profs. Davis, Peck and Dunster, who were to report at the afternoon session.

At 12 o'clock a recess was taken until 3 P. M.

### AFTERNOON SESSION.

Pursuant to adjournment, the Association met again at 3 o'clock.

Prof. Davis, of the committee to whom was referred the resolution offered by Prof. Flint, reported in favor of the resolution with a slight modification, and the same was adopted, as follows:

*Resolved*, That the Secretary of the Association be and he is hereby directed to furnish once in each year to each and every college, members and affiliated colleges the diplomas and tickets of which may be recognized by college members and affiliated colleges; and also to college members and affiliated colleges a printed list of those colleges (not including irregular colleges) of the United States that have applied for membership and have been rejected or expelled from the Association, the diplomas and tickets of which are not to be recognized by college members or affiliated colleges; and also to furnish with said list of rejected colleges not to be recognized the dates at which said colleges had been expelled from membership of the Association, and after which the diplomas and tickets of said colleges are not to be recognized.

On motion, Profs. Davis, Flint and Gross were appointed by the Chair a committee to consider the whole matter in relation to the classification of the Medical Colleges.

Prof. Gross offered a series of preambles and resolutions contemplating a meeting in September of representatives at Washington, for the purpose of raising the uniform standard of medical education.

Prof. Davis seconded the resolution. He referred to the efforts made to improve college instruction during the past twenty-five years, and thought that sentiment had reached a point which demanded an advancement in medical education. He favored a three years' course of instrnction, of not less than eight months' duration per year, and no student could enter upon his studies in in any college until he had first given some evidence of preparation. He hoped to live to see the medical profession at the head of all science, where it belonged, and the adoption of the resolutions would be an important step in the right direction.

Prof. Gunn, of Rush College, was in favor of a three years' course as to the requisite graduation of students.

The Secretary read a letter from Prof. Seely, of the Ohio Medical College, in which he spoke in favor of a full course. The Secretary also stated that he was of the opinion from the correspondence he had had that a majority of the colleges were in favor of a full term.

Prof. Bodine spoke in favor of a three years' course, but thought the time was not suitable. He offered an amendment to the effect that the conference be held under the auspices of the Association, which was lost. The Friday preceding the meeting of the American Medical Association next year was fixed as the day for the holding of the Conference, and the preamble and resolutions were adopted.

Prof. Flint offered a preamble and resolution to the effect that the tickets and diplomas of the Nashville Medical College shall not be recognized by the Association so long as the institution gives two graduating courses a year, and accepts three years' practice in lieu of a course of lectures.

The following named officers were elected for the ensuing year:

President—Prof. J. S. Biddle.
Vice-President—Prof. N. S. Davis.
Secretary and Treasurer—Prof. L. Connor.

Dartmouth Medical College offered its resignation as an active member of the Association. Laid on the table for one year. The college at Fort Wayne, Ind., having been dissolved and a new one founded, the application of the latter to the place of the former was refused, and the new institution directed to send in its formal application as a new college. The College of Physicians and Surgeons of Indianapolis and the Medical Department of the University of Missouri signified their intention to apply for membership. Thanks were tendered to the retiring officers for efficient services, and to the Faculty of the Buffalo Medical College for the use of rooms.

The Association then adjourned to meet at the call of the President.

